# Molecular Characteristics and Pathogenicity Analysis of Bovine Viral Diarrhea Virus Strain Isolated from Persistently Infected Cattle

**DOI:** 10.3390/ani16010153

**Published:** 2026-01-05

**Authors:** Jiaxing Zhong, Fen Sun, Ming Zhou, Kaiqiang Fu, Hongjun Yang

**Affiliations:** 1 Institute of Animal Reproductive Physiology and Disease, College of Veterinary Medicine, Qingdao Agricultural University, Qingdao 266109, China; 2 Shandong Key Laboratory of Animal Disease Control and Breeding, Institute of Animal Science and Veterinary Medicine, Shandong Academy of Agricultural Sciences, Jinan 250100, China; fensun2017@126.com (F.S.);

**Keywords:** bovine viral diarrhea virus, HB2411 strain, pathogenicity, E2 protein, cattle

## Abstract

Bovine viral diarrhea virus (BVDV) primarily causes *bovine* viral diarrhea/mucosal disease, an infectious disease having a significant economic impact on the cattle-farming industry globally. Comprehensive monitoring and in-depth studies of the pathological characteristics of viruses are crucial in formulating effective prevention and control strategies. The isolation, identification, molecular characterization, and pathogenicity analysis of a BVDV strain isolated from persistently infected *cattle* ear tissue samples are reported in this study. This newly isolated strain is a noncytopathogenic BVDV, which we named HB2411. Homology between the HB2411 and U63479 strains was determined to be 96.7%, and the phylogenetic tree indicated that HB2411 belongs to the BVDV-1b subtype. Genetic variation analysis of the E2 protein of the HB2411 strain revealed multiple amino-acid mutation sites. Recombination analysis of the newly isolated HB2411 strain suggested a potential cross-geographical transmission event. *BALB/c mice* were intraperitoneally inoculated with the BVDV strain to evaluate the pathogenicity and virulence of BVDV-1b HB2411. BVDV was detected in multiple organs of *BALB/c mice*, with the highest viral load in the liver. BVDV infection promoted the expression of inflammatory cytokines in mice livers, necessitating further studies on the virulence and pathogenic mechanisms of this new strain to reduce economic losses caused to the animal husbandry industry.

## Simple Summary

Bovine viral diarrhea is a contagious disease caused by infection with the bovine viral diarrhea virus, one of the major infectious diseases that seriously threaten the global cattle industry. BVDV belongs to the genus Pestivirus within the family Flaviviridae and can infect various domestic animals, including cattle. In this study, a BVDV strain was isolated and identified from a cattle herd in Hebei Province, China. The complete genome of the newly isolated strain was subjected to phylogenetic analysis, recombination analysis, and sequence analysis of the E2 region. Furthermore, its pathogenicity was evaluated in BALB/c mice.

## 1. Introduction

*Bovine* viral diarrhea (BVD)/mucosal disease (MD) is an important infectious disease in cattle caused by the BVD/MD virus. BVD virus (BVDV) is a single-stranded RNA virus with a genome of approximately 12.5 kb that belongs to the Flaviviridae family and Pestivirus genus. It is one of the most common pathogens in the *cattle* industry globally [1]. BVDV infection can lead to multisystem dysfunction in *cattle*, including diseases of the digestive, respiratory, and reproductive systems [2]. Acute infections may result in congenital malformations and the development of calves with persistent infection (PI), which acquire BVDV immune tolerance through vertical viral transmission during early pregnancy [3]. As animals with PI shed large amounts of the virus throughout their lifespan, they are the primary source of infection among susceptible animals, maintaining the infection cycle [4] and making them key targets in controlling disease transmission [5]. Currently, there are no specific effective treatments for this disease, and prevention relies primarily on vaccination. However, due to the complex serotypes, frequent mutations, and strong transmission ability of the pathogen, existing measures of control still fail to achieve ideal results [6]. BVDV exhibits high genetic diversity. Based on partial gene or genome sequences, BVDV can be classified into BVDV1, BVDV2, and the newly emerged HoBi-like virus (BVDV3). The main circulating strains in China belong predominantly to the BVDV1 type [7].

BVDV can be classified into cytopathic (CP) and noncytopathic (NCP) biotypes [8]. CP strains result from marked genetic changes in the lethal form of BVDV, arising from NCP viruses that mutate into CP in animals with PI. Numerous in vitro studies have shown that only the NCP biotype of BVDV can establish and maintain PI [9]. The BVDV genome is approximately 12.3 kb in length and contains a single open-reading frame (ORF) flanked by 5′ and 3′ untranslated regions (UTRs). The 5′ end of BVDV includes an internal ribosome entry site that facilitates the translation of a single polyprotein. The 3′ end contains a conserved stem-loop structure instead of a poly-A tail and has binding sites for various host cell microRNAs [10,11]. The ORF of BVDV encodes 11–12 proteins, including Npro, C, Erns, E1, E2, P7, NS2–3 (or NS2 and NS3), NS4A, NS4B, NS5A, and NS5B, in the order from 5′ to 3′. Among these, C is the capsid and structural protein, whereas Erns, E1, and E2 are envelope proteins, with E2 being the major neutralizing antigen [12].

E2 is the most abundant protein on the surface of virus particles that can strongly induce immune responses against BVDV [13]. Studies on its crystal structure indicate that it consists of two immunoglobulin (Ig)-like domains and a folded and elongated β-chain structure, forming a covalently linked dimer [14]. The conserved C-terminal region at the connection site is abundant in aromatic residues. Both the I and II structural domains on the viral surface have been found to induce immune responses using antigen epitope peptide prediction tools, and potential epitopes have been reported to likely exist in domain III. This structural characteristic not only enhances the antigenicity of E2 but also contributes to its other functions including membrane fusion and viral entry [15]

Isolation of the pathogen from the ear tissues of calves with diarrhea in a large-scale dairy farm in Hebei Province and its identification were undertaken in this study. The main pathogen responsible for calf diarrhea was determined, and the cell-infection ability, whole-genome sequence, and genetic evolution of the pathogen were analyzed. A mouse model of infection was established in *BALB/c mice* to determine the pathogenicity of the novel strain. Our findings provide new evidence identifying the main pathogen of calf diarrhea in China, offer insights into the epidemiology and control of BVDV, and foundational data for mechanistic studies and vaccine development targeting pathogenic BVDV.

## 2. Materials and Methods

### 2.1. Cell Culture and Virus Isolation

MDBK (the MDBK cell line was obtained from Cell Bank, Type Culture Collection, Chinese Academy of Sciences, Beijing, China, GNO7) cells were cultured in DMEM containing 10% FBS. The samples were obtained from a dairy farm in Hebei, where cows showed clinical symptoms such as diarrhea and abortion, indicating a potential BVDV infection. One ear biopsy sample was collected from the diarrhea-affected *cow* and tested positive for BVDV antigens via PCR.

For virus isolation, 4 mL of phosphate-buffered saline (PBS, pH = 7.2) was added to the ear biopsy samples, followed by vortexing for 1 min. The samples were then centrifuged at 12,000 rpm at 4 °C for 10 min, and the supernatant was collected. In a biosafety cabinet, the supernatant was transferred into a sterile, autoclaved 1.5 mL centrifuge tube and filtered through a 0.22 μm membrane filter to remove contaminants. The filtered supernatant was inoculated onto MDBK cells grown to a monolayer. The cells were then incubated in a 37 °C incubator with 5% CO_2_ for 1 h. After incubation, the inoculum was discarded, and the cells were maintained with DMEM (gibco, 11965092) containing 2% FBS (gibco, A5256701). The culture was then continued in the incubator, and cytopathic effects (CPE) were monitored daily.

After 72 h, the cells were collected and subjected to three freeze–thaw cycles at −80 °C. The cells were then centrifuged at 8000 rpm for 5 min to remove cell debris, and the supernatant was collected and passaged onto MDBK cells for the next round of viral cultivation. Three consecutive viral passages were performed, with PCR conducted at each passage. BVDV strain samples stored at −80 °C. The BVDV-1 AV69 strain was obtained from the China Institute of Veterinary Drug Control.

### 2.2. Indirect Immunofluorescence Assay (IFA)

MDBK cells were seeded in a 6-well plate and allowed to grow until the cell density reached approximately 80%. The culture medium was discarded, and the cells were washed three times with PBS. After discarding the wash solution, 1 mL of DMEM containing double serum was added to each well. The collected virus supernatant was inoculated at 200 µL per well, with three replicates and three negative control groups. The 6-well plate was placed in a 37 °C incubator with 5% CO_2_ and incubated for 2 h. After the incubation, the inoculum was discarded, and 2 mL of DMEM containing 2% FBS was added to each well. The plate was then placed back in the incubator for continued culture for 72 h.

After 72 h of virus infection, the culture medium was aspirated, and approximately 200 µL of wash buffer (PBS, pH 7.2) was gently added to each well for a single wash. The buffer was then aspirated, and 200 µL of pre-cooled fixative was immediately added to each well for 15 min fixation. Following aspiration and air-drying in a biosafety cabinet, each well was washed once with 200 µL wash buffer and drained. Diluted BVDV primary antibody (1:50 using the antibody diluent provided in the kit) was added at 100 µL per well and incubated at 37 °C for 60 min. The antibody was aspirated, and wells were washed three times with 200 µL wash buffer per wash and drained. Diluted FITC-conjugated secondary antibody (1:50 using the kit diluent) was then added at 100 µL per well and incubated at 37 °C for 60 min. Wells were washed three times with 200 µL wash buffer per wash, drained, and finally 100 µL of wash buffer was added to each well. Fluorescence was observed under a fluorescence microscope.

### 2.3. 50% Tissue Culture Infectious Dose (TCID50) Analysis

During the virus blind passage, no typical cytopathic effect was observed. Therefore, TCID50 determination was performed using Immunofluorescence Assay (IFA). After passing the virus through three consecutive passages. MDBK cells were seeded in a 96-well cell culture plate and cultured until the cell density reached approximately 70%. Then, 100 µL of BVDV suspension was added to 900 µL of DMEM, followed by a serial 10-fold dilution. The diluted virus was inoculated onto the 96-well plate, with 100 µL per well, and 8 replicates were set for each dilution. A 100 µL of DMEM was used as a blank control. The plate was incubated at 37 °C with 5% CO_2_ for 72 h. After incubation, the number of wells with infected cells was detected using a fluorescence microscope. The dilution factor was recorded, and the virus TCID50 was calculated using the Reed-Muench method.

### 2.4. RNA Extraction and RT-PCR

According to the manufacturer’s instructions, RNA was extracted from the infected cell lysate using the Fast Pure Viral DNA/RNA Mini Kit (Vazyme, Nanjing, China).

For reverse transcription, the Evo M-MLV Reverse Transcription Premix Kit (with gDNA Removal, for qPCR) Ver.2 (AG, Changsha, China) was used to synthesize cDNA. PCR amplification was performed using 2 × Flash HS PCR Master Mix (AG, Changsha, China) in a 50 µL reaction volume, containing 25 µL of 2 × Flash HS PCR Master Mix, 2.5 µL of both upstream and downstream primers, 2 µL of cDNA template, and 18 µL of ddH_2_O. The PCR program included an initial denaturation at 95 °C for 2 min, followed by 35 cycles of 15 s at 95 °C, 15 s at 58 °C, and 15 s at 72 °C, with a final extension at 72 °C for 5 min.

### 2.5. Transmission Electron Microscope

After performing three cycles of freeze–thawing on the cell culture supernatant, the sample was collected and centrifuged at 10,000 rpm at 4 °C for 30 min to remove the majority of the cell debris and impurities. The supernatant was then collected and further concentrated by centrifuging at 35,000 rpm for 4 h. The supernatant was discarded, and the resulting pellet was resuspended in 100 µL of PBS. A 10 µL aliquot of the suspension was placed onto a copper grid for electron microscopy, where it was adsorbed at room temperature for 10 min. The grid was then stained with 3 µL of 2% phosphotungstic acid for 90 s, followed by natural air drying at room temperature. The viral particles were observed under a transmission electron microscope.

### 2.6. Virus Copy Number Determination

MDBK cell suspension (2 mL per well) was seeded into a 6-well plate and cultured for 24 h until a monolayer was formed. The culture medium was then discarded, and the virus was inoculated (MOI = 1). The plate was placed in a 37 °C incubator with 5% CO_2_ for 2 h to allow adsorption. Afterward, the inoculum was discarded, and the cells were washed three times with PBS. Next, 2.5 mL of cell maintenance medium was added to each well, and the cells were incubated further. This time point was designated as 0 h. Virus supernatant was collected at the following time points: 0 h, 12 h, 24 h, 36 h, 48 h, 60 h, 72 h, 84 h, and 96 h. For each time point, three replicates were performed. The collected virus supernatants were subjected to nucleic acid extraction, followed by virus copy number determination.

### 2.7. Whole Genome Sequencing and Sequence Analysis of the Virus

Sequence alignment of the isolated BVDV strain and known BVDV strains was performed using MEGA 11 software. Phylogenetic analysis was conducted using MEGA 11 software to assess the genetic relationships between the isolated strain and other BVDV strains. The three-dimensional structure of the BVDV E2 protein was predicted using AlphaFoldsoftware (AlphaFold 3). To investigate possible gene recombination events, SimPlot software (version 3.5.1) was employed to analyze the genetic recombination between the newly isolated strain and reference BVDV strains.

### 2.8. Animal Experiment and Sample Collection

A total of 21 *BALB/c mice*, aged 6–7 weeks, were purchased from Shandong Pengyue Laboratory Animal Science and Technology Co., Ltd. (Jinan, China). After a 1-week isolation period, the mice were observed for normal physiological status. The mice were housed in specialized ventilated cages, with sufficient water and food provided throughout the feeding period. The mice were randomly divided into a virus infection group and a control group. The virus infection group was intraperitoneally injected with 200 µL of a 10^6^ TCID50 virus solution, while the control group received the same volume of DMEM.

*Mice* were monitored daily for behavior and mental status. On days 3, 4, 7, 8, 9, and 10 post-infection, mice were euthanized by cervical dislocation, and the heart, liver, spleen, lungs, and kidneys were collected and stored at −80 °C until further analysis. After weighing each organ, they were homogenized into tissue homogenates, and total RNA was extracted using Trizol Reagent. The RNA was reverse transcribed into cDNA, which served as the template for the mRNA expression analysis. The expression levels of BVDV mRNA in the organs of the mice were measured using ChamQ Universal SYBR qPCR Master Mix (Vazyme, Nanjing, China, Q711-02). Additionally, the mRNA levels of inflammatory cytokines, including *IL-6*, *IL-10*, *TNF-α*, and *IFN-α*, were assessed in the liver tissue of the mice by RT-qPCR.

### 2.9. H&E Staining

Tissue sections were prepared according to standard operating procedures (SOPs) for pathological sampling, fixation, embedding, and sectioning, including paraffin-embedded and frozen sections. For paraffin sections, dewaxing to water was performed by sequentially immersing the slides in Eco-friendly Dewaxing Solution I for 20 min, Dewaxing Solution II for 20 min, absolute ethanol I for 5 min, absolute ethanol II for 5 min, and 75% ethanol for 5 min, followed by rinsing with tap water. For frozen sections, slides were equilibrated from −20 °C to room temperature and fixed with tissue fixative for 15 min, followed by rinsing in running water. Pretreatment was conducted by incubating the sections in a high-definition hematoxylin pretreatment solution for 1 min. Hematoxylin staining was performed by immersing the sections in hematoxylin solution for 5 min, rinsing with tap water, differentiating with differentiation solution, rinsing again, bluing in bluing solution, and washing under running water. Eosin staining was performed by dehydrating the slides in 95% ethanol for 1 min and staining in eosin solution for 15 s. Dehydration and mounting were performed by sequentially immersing the slides in absolute ethanol I (2 min), absolute ethanol II (2 min), absolute ethanol III (2 min), n-butanol I (2 min), n-butanol II (2 min), xylene I (2 min), and xylene II (2 min) for clearing, followed by mounting with neutral resin. Microscopic examination was conducted, and images were captured and analyzed.

### 2.10. Heparin Sodium Competitive Inhibition Assay

MDBK cells were seeded in 6-well plates and used when the cell confluence reached approximately 80%. Heparin sodium (0 or 50 mg/mL) was mixed with 1 mL of AV69 or HB2411 viral suspension, and the heparin sodium–virus mixtures were then inoculated onto MDBK cells. After incubation at 37 °C for 1 h, the inoculum was removed and replaced with DMEM maintenance medium supplemented with 2% FBS. Viral copy numbers were quantified at 24 h post-infection.

### 2.11. Neutralization Assay

The HB2411 isolate and the reference strain AV69 were serially diluted tenfold (10^−1^ to 10^−8^). Each virus dilution was mixed with an equal volume of positive serum that had been heat-inactivated at 56 °C for 30 min. The virus–serum mixtures were incubated at 37 °C in a water bath for 60 min, with vortexing for 10 s every 5 min to ensure thorough mixing. In parallel, control groups were established by inoculating MDBK cells with HB2411 or AV69 virus suspensions diluted tenfold from 10^−1^ to 10^−8^ without serum. Pre-prepared PLT cells seeded in 96-well plates were used, and 100 μL of each virus–serum mixture was added per well. For each dilution, six replicate wells were included to assess assay reproducibility, and wells containing only cell culture medium served as cell controls. After incubation at 37 °C for 72 h, the number of fluorescent-positive wells was determined by immunofluorescence assay (IFA). The 50% tissue culture infectious dose (TCID_50_) for each group was calculated using the Reed–Muench method. The neutralization index (NI) was calculated as the difference between the logarithmic virus titers of the neutralization groups and the corresponding control groups.

## 3. Results

### 3.1. Isolation and Identification

**Figure 1:**
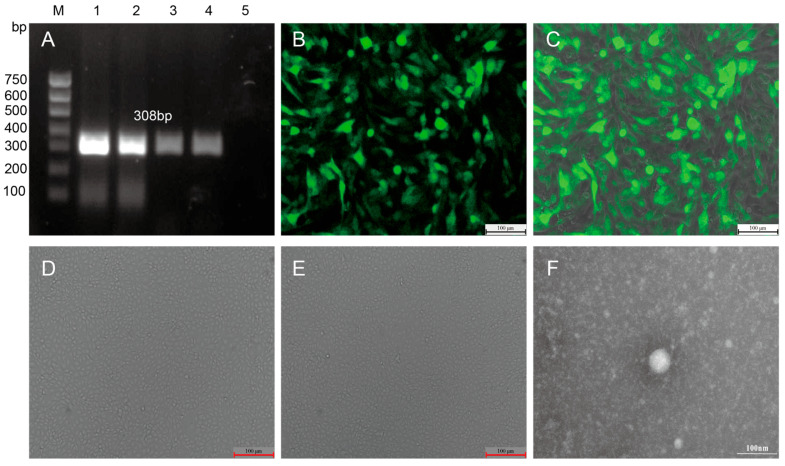
BVDV strain isolated from an ear tissue sample of a cow with diarrhea. (**A**). Amplification signals with 308 bp were detected in all MDBK cells infected with the ear tissue sample using RT-PCR. M. DNA Marker (D750); 1-4. BVDV HB2411 infected cells; 10. Negative Control. (**B**). Characteristic green fluorescence signals, indicative of viral infection, were observed in cells by IFA. (**C**). Merged image (fluorescence + bright field) shows infected cells. (**D**). BVDV-infected MDBK cells. (**E**). BVDV-negative MDBK cells. (**F**). Electron microscopy examination of purified MDBK cell cultures harvested 72 h post-BVDV inoculation revealed numerous viral particles with characteristic Pestivirus morphology.

After processing cow ear-tissue samples cultured in MDBK cells, RT-PCR and immunofluorescence techniques were used to determine virus replication. Findings from RT-PCR showed an expected band of 308 bp in all infected cells (Figure 1A). Immunofluorescence analysis revealed significant and specific green fluorescence signals in the cytoplasm of infected cells (Figure 1B,C), confirming effective replication of BVDV in MDBK cells. After three consecutive generations of stable virus passage, a purified BVDV strain, named HB2411, was successfully isolated.

To further characterize the biological properties of this strain, MDBK cells infected with the HB2411 strain (experimental group) and uninfected cells (control group) were cultured under similar conditions for 72 h, followed by systematic observation using microscopy. Neither group of cells exhibited typical CP effects (Figure 1D,E), and the cell morphology, growth density, and proliferation status were almost identical, indicating the isolated HB2411 strain to be an NCP biotype, which was consistent with the characteristics of most circulating BVDV strains.

Findings from electron microscopy revealed numerous typical BVDV particles clearly visible in the cytoplasm of infected cells (Figure 1F). These particles exhibited a regular spherical or near-spherical structure, with a complete lipid envelope and a diameter ranging from 50–100 nm, matching the typical viral particles of the Pestivirus genus. Furthermore, the ultrastructure of the virus particles closely matched the morphological characteristics of BVDV reported in the literature, further confirming the accuracy of the isolated virus from a morphological perspective.

### 3.2. 50% Tissue Culture Infectious Dose

After diluting the isolated strain HB241 by a factor of 10, it was inoculated into MDBK cells and cultured for 72 h. The cells were fixed with 4% paraformaldehyde, and viral infection was determined based on immunofluorescence. The viral titer of the isolated strain was calculated using the Reed–Muench method, resulting in a titer of 10–6.5 TCID50/mL.

### 3.3. Copy Number

**Figure 2:**
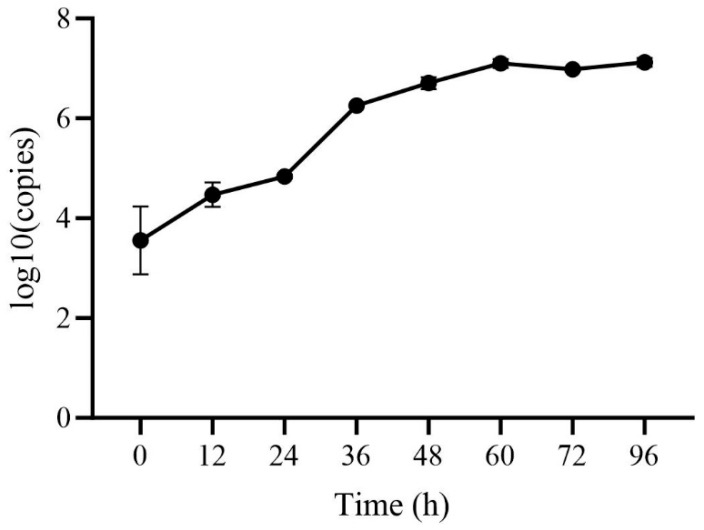
Time-course dynamics of HB2411 strain viral load quantified by RT-qPCR.

The proliferation kinetics of the HB2411 strain demonstrated a typical S-shaped growth curve (Figure 2). A noticeable increase in copy number was detected within 12 h after infection, indicating the short incubation period of the strain. The virus entered the logarithmic growth phase during the 24–36 h period, with the copy number increasing exponentially and reaching the maximum proliferation rate, suggesting the phase to be the critical window for viral replication. As the infection time increased, the viral-replication rate gradually decreased after 60 h, eventually reaching a relatively stable plateau phase. These dynamic changes followed the fundamental pattern of viral proliferation.

### 3.4. Homology Analysis

**Figure 3:**
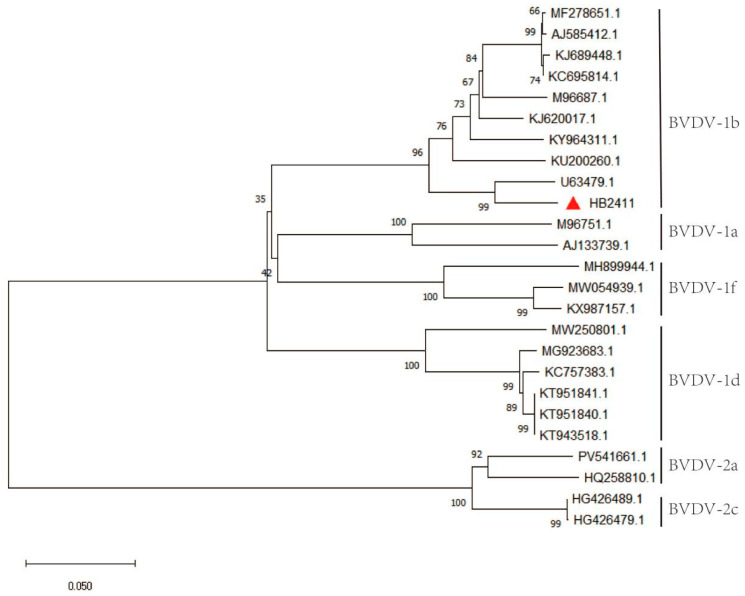
Phylogenetic tree construction of BVDV based on whole-genome sequences and the red triangle was HB2411.

**Figure 4:**
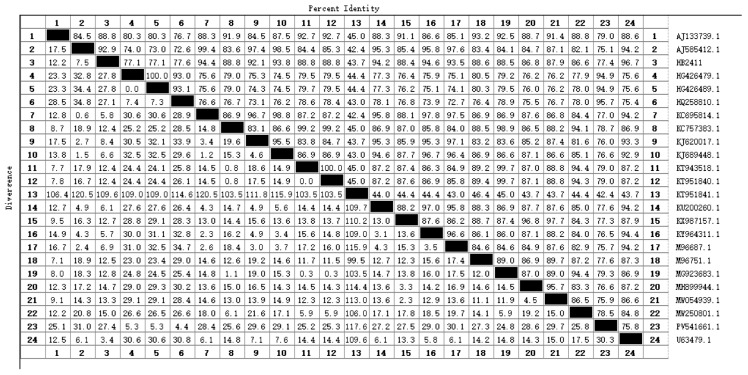
Nucleotide sequence alignment of HB2411 stain.

The full genome length of the HB2411 strain is 12,292 nucleotides, with a CG content of 45%. The genome sequence includes a 369-nucleotide 5′ UTR, a 288-nucleotide 3′ UTR, and an ORF encoding a precursor polyprotein of 3898 amino acids. To determine the genetic evolutionary characteristics of the BVDV HB2411 strain, a homology comparison of its 5′ UTR sequence was performed with reference strains of different genotypes from GenBank. Sequence alignment was conducted using the MUSCLE algorithm in MEGA 11 software, and a phylogenetic tree was constructed using the neighbor-joining method. Phylogenetic analysis indicated that the HB2411 strain clustered in the same evolutionary branch as the BVDV-1b strains, confirming its classification as the BVDV-1b subtype (Figure 3). The 5′ UTR sequence of the BVDV HB2411 strain was found to share the highest homology (96.7%) with the BVDV reference strain 1b (U63479.1), with an average homology of 94.03%. In contrast, homology with other genotype strains was lower, with an average nucleotide homology of only 82.33% (Figure 4). Further analysis revealed HB2411 to be most closely related to the strain U63479.1 reported in New York, forming an independent small branch.

### 3.5. E2 Protein Amino Acid Sequence Analysis

**Figure 5:**
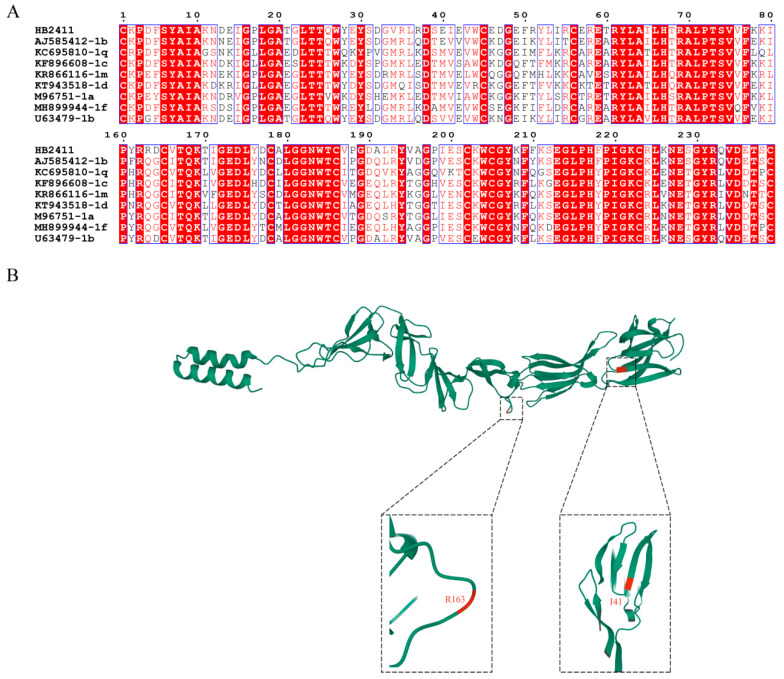
Amino acid characteristics of E2 protein. (**A**) Amino acid sequence alignment of E2 proteins of the nine new BVDV isolates and 16 reference isolates. Amino acid deletions or mutations of these isolates were shadowed and described in detail in the text. (**B**) Tertiary structure on the E2 protein of the new isolates. The unique amino acid residues are marked in red.

Glycoprotein E2 plays a crucial role in immune function, viral pathogenicity, and cell entry. To confirm the important glycoprotein variations in the BVDV isolate HB2411, the E2 protein amino acid sequences of the HB2411 strain were compared with those of the other BVDV reference strains (Figure 5A). The red-marked regions represent highly conserved amino acid areas, whereas the unmarked regions indicate differences in amino acids. Compared with other strains, the E2 protein of the HB2411 strain exhibits 2 unique amino acid mutation sites (I41, R163) (Figure 5B). These mutations involve the substitution of valine with isoleucine at position I41 and the mutation of glutamine to arginine at position R163. The latter represents a shift from a neutral to a basic amino acid, which is a highly significant nonconservative mutation. Glutamine is a polar neutral amino acid, whereas arginine is a positively charged basic amino acid. This mutation fundamentally alters the charge properties and side chain structure of amino acids at this position.

### 3.6. Gene Recombination Analysis

**Figure 6:**
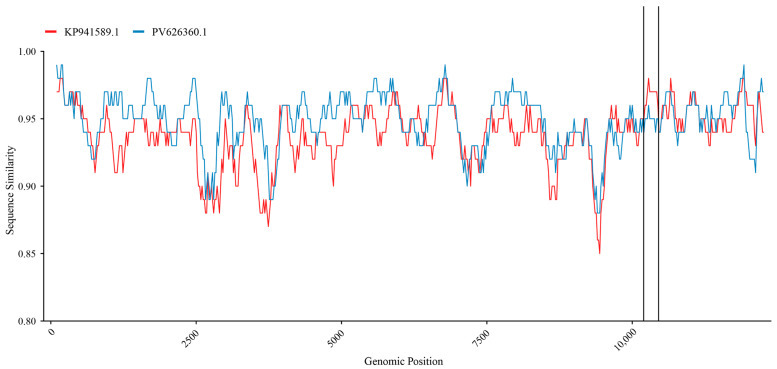
Recombination analysis on the genomes of the new BVDV isolates.

Gene recombination analysis of the full-genome sequences of the isolated strain and reference strains was performed using SimPlot software (version 3.5.1). Recombination signals were identified in the nucleotide sequence region around positions 10,221–10,441 (Figure 6). The PV626360.1 strain was identified as the primary parent of HB2411, whereas the KP941589.1 strain was identified as the secondary parent.

### 3.7. Messenger RNA (mRNA) Expression of BVDV in Different Tissues of Mice

**Figure 7:**
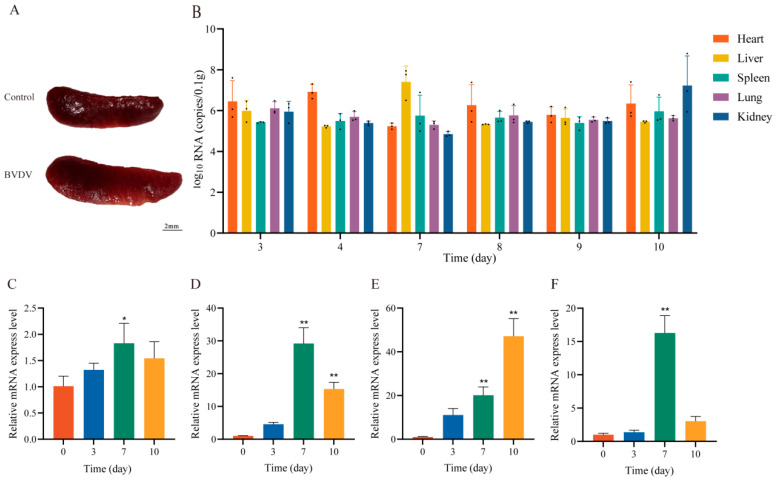
Post-infection tissue examination in BVDV-challenged mice. (**A**). Comparison of Mouse Spleens. (**B**). Viral load of BVDV in various mouse tissues. The mRNA expression levels of *IL-6*, *IL-10*, *TNF-α* and *IFN-α* in mouse liver; (**C**). *IL-6*; (**D**). *IL-10*; (**E**). *TNF-α*; (**F**). *INF-α*.*: *p* < 0.05, **: *p* < 0.01.

BVDV-infected mice exhibited significant splenomegaly compared with control mice (Figure 7A). Findings from RT-qPCR indicated that after experimental mice were infected with BVDV, the virus was detected in multiple tissues and organs, including the heart, liver, spleen, lungs, and kidneys, in the experimental group of mice on days 3, 4, 7, 8, 9, and 10 (Figure 7B). The viral load exhibited a dynamic change trend, with the most significant infection observed in liver tissues. The peak viral load reached 10^7.4^ copies/mg on post-infection day 7.

### 3.8. BVDV Promotes the Expression of Inflammatory Cytokines in Mice

We designed primers targeting inflammatory cytokines (Table 1). RT-qPCR revealed that the expression of cytokines in the livers of BVDV-infected mice exhibited significant time-dependent changes. The mRNA expression of *IL-6* in the livers of mice in the infected group increased significantly on day 7 compared with that in control mice (Figure 7C). Meanwhile, the mRNA expression of *IL-10* increased significantly on day 7 (Figure 7D), indicating that the viral infection may have induced an anti-inflammatory response. Notably, the mRNA expression of the pro-inflammatory factors tumor necrosis factor *(TNF)-α* and interferon *(IFN)-α* remained higher than that noted in the control group on days 3, 7, and 10 post-infection, with differences on days 7 and 10 being highly significant (Figure 7E,F). These results suggest that the viral infection triggered a significant inflammatory response, which peaked on day 7 post-infection and continued until day 10. Dynamic changes in the cytokine expression profile suggest that the viral infection may have influenced disease progression by modulating the balance between the pro- and anti-inflammatory factors.

### 3.9. Hematoxylin and Eosin Staining

**Figure 8:**
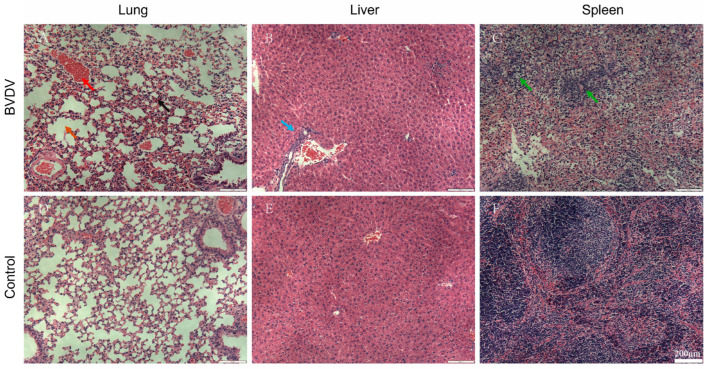
Pathologic changes in various organs of mice (400×). Scale bar is 200 µm. (**A**) Lung tissue slice from the BVDV group. (**B**) Liver tissue slice from the BVDV group. (**C**) Spleen tissue slice from the BVDV group. (**D**) Lung tissue slice from the control group. (**E**) Liver tissue slice from the control group. (**F**) Spleen tissue slice from the control group. The number in the lower right corner indicates a magnification of 200×. The red arrows indicate congestion; the black arrows indicate hemorrhage; the brown arrows indicate alveolar rupture; the blue arrows indicate inflammatory cell infiltration; and the green arrows indicate an indistinct boundary between the red and white pulp of the spleen. The scale bar represents 200 μm.

Histopathological examination of the tissues of the organs of mice revealed significant findings. As shown in Figure 8, the liver tissues in the BVDV group exhibited notable inflammatory cell infiltration. The boundary between the red and white pulp of the spleen was blurred. The tissue structure was unclear and was accompanied by inflammatory edema and a large number of macrophages. Severe hemorrhage and congestion, as well as rupture of the alveolar walls leading to a loss of structural integrity, were observed in lung tissues.

### 3.10. Heparin Sodium Inhibits HB2411 Infection of MDBK Cells

**Figure 9:**
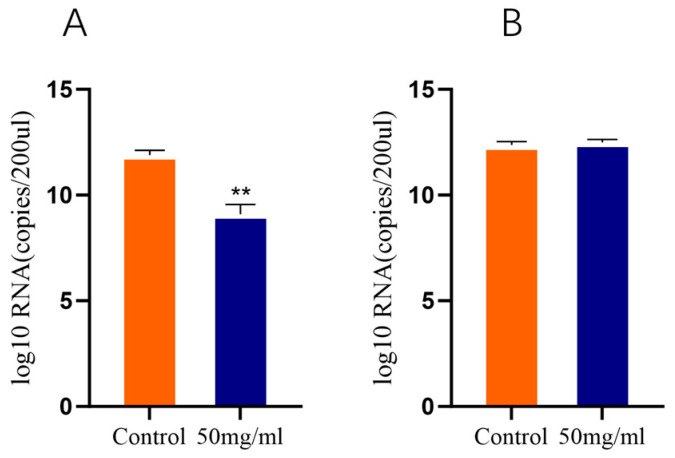
Replication kinetics of BVDV strains AV69 and HB2411. Effects of 50 mg/mL Heparin Sodium on Viral Copy Numbers in Infected MDBK Cells at 24 Hours Post-Treatment. (**A**). HB2411 strain (**B**). AV69 strain. **: *p* < 0.01.

As shown in Figure 9A, at 24 h post-infection of MDBK cells with BVDV, treatment with 50 mg/mL heparin sodium significantly reduced the HB2411 viral copy number compared with the control group, whereas no significant effect was observed on AV69 (Figure 9B).

### 3.11. Neutralizing Activity of Positive Serum Against the Isolated Virus

**Figure 10:**
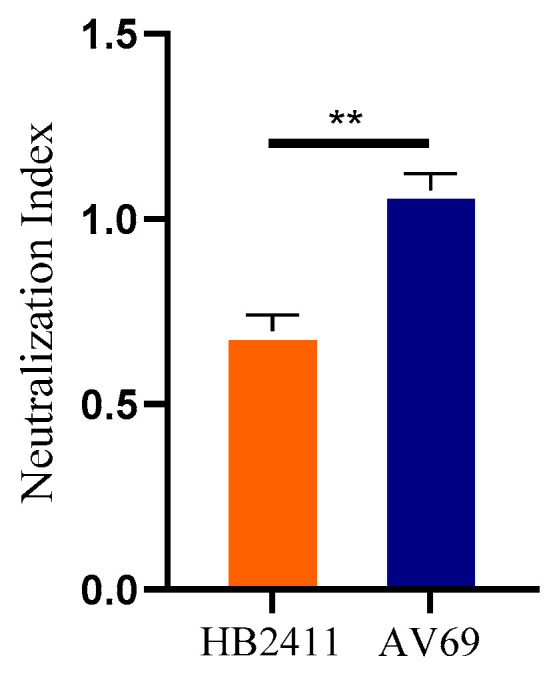
Neutralization indices of the HB2411 and AV69 strains. **: *p* < 0.01.

The neutralizing capacity of positive serum against the HB2411 isolate and the reference strain AV69 was determined by a virus neutralization assay. As shown in Figure 10, the neutralization index (NI) of the HB2411 isolate was 0.697, indicating a relatively low neutralization efficiency of the positive serum against its viral titer, whereas the NI of the AV69 strain was 1.08.

## 4. Discussion

BVDV shows a wide distribution globally. The clinical symptoms of BVDV-infected cattle include diarrhea, fever, mucosal ulceration and necrosis, and decreased reproductive performance. This disease is also known as bovine viral diarrhea MD [16]. Molecular epidemiological surveys have confirmed that BVDV-1b is the predominant subtype circulating in cattle populations in northern China. For instance, in Heilongjiang Province, it accounts for over 47% of positive samples. This dominant status establishes it as the primary target for regional prevention and control strategies [17]. As a positive-sense RNA virus, BVDV possesses an RNA polymerase that lacks proofreading functionality, making errors frequent during replication. When the same host cell is co-infected with two distinct strains of BVDV, the viral RNA genomes may undergo template switching, resulting in recombinant viruses that incorporate genetic segments from both parental strains. In this study, a BVDV strain was successfully isolated using the MDBK cell line. Its molecular characteristics were consistent with those of previously reported strains, and the virus was confirmed to belong to the NCP type. Using whole-genome sequencing, the strain was confirmed as BVDV-1b, which is highly similar to a BVDV-1b strain from Germany. BVDV was first isolated in the United States and was later reported worldwide [18]. The predominant BVDV genotype that is currently circulating in China is Type 1 [19], Similarly, in our study, viral RNA was identified in most tissues and organs of infected mice, with varying viral loads observed across different tissues. The highest viral load was detected in the liver on day 7 post-infection. The hepatocyte membrane contains a variety of potential viral adsorption factors and entry receptors, and some receptors (such as heparan sulfate proteoglycans and members of the low-density lipoprotein receptor family) are highly expressed in the liver. Specific amino acid mutations in viral envelope glycoproteins (such as E2) may optimize the affinity or specificity of viral particles binding to these receptors, enabling the virus to infect hepatocytes more efficiently and ultimately achieve higher levels of replication in the liver. In this study, the Q163R mutation may directly contribute to hepatic tropism. HB2411 and AV69 strains were treated with 50 mg/mL heparin sodium. After 24 h, the copy number of the HB2411 strain was detected to be significantly lower than that in the control group (Figure 9A,B). Despite the spleen being an organ that is active in immune responses, the unique immune-tolerant microenvironment of the liver [20] may further promote continuous viral replication. Yet, the higher viral load in the liver might be related to its immune-evasion mechanisms.

The host immune system involves various cytokines for antiviral responses and immune regulation during a BVDV infection [21]. Among these, type I *IFN-α* is a core factor in antiviral immunity. It inhibits viral replication by activating the JAK-STAT pathway and inducing the expression of *IFN*-stimulated genes [22]. Studies have shown that the antiviral activity of IFN-1 is related to the inhibition of cholesterol and fatty acid synthesis [23]. Meanwhile, the levels of pro-inflammatory factors, including *TNF-α* and *IL-6*, increase significantly during acute infections. *TNF-α* clears the virus by activating macrophages and inducing cell apoptosis, whereas *IL-6* participates in acute-phase inflammation [24]. An increasing number of clinical trials show that the immune response of the body involves the secretion of large amounts of *IL-6* to counteract the viral infection, thereby promoting disease progression [25]. *IL-6* expression in the liver peaked on day 7, coinciding with the highest viral load in the liver at the same time. This result is also consistent with observations in hepatitis B virus infections. Notably, BVDV infection induces the expression of the anti-inflammatory cytokine *IL-10* [26]. As a key immune-regulatory factor, *IL-10* regulates both Th1 and Th2 immune responses via a negative feedback mechanism and significantly inhibits the production of the pro-inflammatory cytokines *IL-6* and *TNF-α* [27]. The abundant endoplasmic reticulum in the unique immune microenvironment of the liver provides favorable conditions for viral replication [28], whereas the high local expression of *IL-10* may further help the virus escape immune clearance [29]. The dynamic balance of these cytokines determines the outcome of BVDV infections. *IL-10* plays a key role in the immune-evasion mechanism of porcine reproductive and respiratory syndrome virus [30]. Similarly, both the expression of *IL-10* and viral load of BVDV in the liver peaked on day 7 post-infection, suggesting the ability of the virus to evade the immune system of the host, thereby increasing the likelihood of persistent infections

BVDV, as an RNA virus, exhibits genetic diversity during its replication and evolution processes [31]. E2 protein is the major surface protein of BVDV that is responsible for binding to host cell receptors and playing a crucial role related to virus entry into cells. In the newly isolated HB2411 strain, the E2 protein exhibits a mutation at position 163, where glutamine is substituted by arginine. The presence of positively charged arginine may significantly enhance the interaction between the virus and the negatively charged molecules, such as heparan sulfate proteoglycans (HSPG) on the host cell surface [32]. Similarly, the Q163R mutation may directly contribute to hepatic tropism, perhaps by facilitating binding to abundant receptors in hepatocytes, which suggest that binding to abundant receptors in hepatocytes is a key area for future research. A comparative analysis in bovine primary hepatocytes revealed that at 48 h post-infection, HB2411 achieved a significantly higher copy number than AV69 (Supplementary Figure S1). Several viruses exploit this electrostatic attraction as the first step in adhesion to the cell surface, which may increase the infection efficiency. E2 protein is also the primary target of the host immune system (neutralizing antibodies), rendering it a major antigen [33]. A mutation from a neutral to a positive charge may alter the surface charge distribution and conformation of this region, helping the virus evade recognition by pre-existing neutralizing antibodies, thereby leading to immune evasion. However, the specific mechanism by which the Q163R mutation promotes the binding of the virus and liver cell receptors still needs further investigation.

## 5. Conclusions

In this study, a novel virus strain was successfully isolated from the ear tissue samples of cattle with diarrhea and named BVDV-HB2411. Phylogenetic analysis revealed that the isolated strain exhibited high nucleotide similarity with the BVDV-1b subtype, indicating the genotype of the BVDV infection in the cattle farm as BVDV-1b. The relative expression of *IL-6*, *IL-10*, *TNF-α*, and *IFN-α* in the livers of infected mice significantly increased after BVDV infection, indicating that the infection triggers an inflammatory response in the host. Our findings contribute to a better understanding of the pathogenicity of BVDV in BALB/c mice. Despite these contributions, our study has some limitations. Future research should focus on exploring the potential mechanisms by which BVDV induces its immunopathological effects.

## Figures and Tables

**Table 1:** Sequences of primers used in the study.

Primer Name	Forward Primer (5′ to 3′)	Reverse Primer (5′ to 3′)
BVDV	GAAGGCCGAAAAGAGGCTC	CATGTGCCATGTACAGCAGAG
*GAPDH*	CATCACTGCCACCCAGAAGACTC	ATGCCAGTGAGCTTCCCGTTCAG
*IL-6*	TACCACTTCACAAGTCGGAGGT	CTGCAAGTGCATCATCGTTGTTC
*IL-10*	CGGGAAGACAATAACTGCACCT	CGGTTAGCAGTATGTTGTCCAGC
*TNF-α*	GGTGCCTATGTCTCAGCCTCTC	GCCATAGAACTGATGAGAGGGAG
*INF-α*	GGATGTGACCTTCCTCAGACTA	ACCTTCTCCTGCGGGAATCCAA

## Data Availability

The original contributions presented in this study are included in the article/Supplementary Material. Further inquiries can be directed to the corresponding authors.

## Supplementary Materials

The following supporting information can be downloaded at https://www.mdpi.com/article/10.3390/ani16010153/s1. Figure S1: Copy numbers of strains HB2411 and AV69 in infected primary liver cells.
