# Supplementary figures and images for: Molecular Characteristics and Pathogenicity Analysis of Bovine Viral Diarrhea Virus Strain Isolated from Persistently Infected Cattle
Source: Animals (Basel). 2026 Jan 5;16(1):153. doi: 10.3390/ani16010153 (PMC12784899; doi:10.3390/ani16010153)

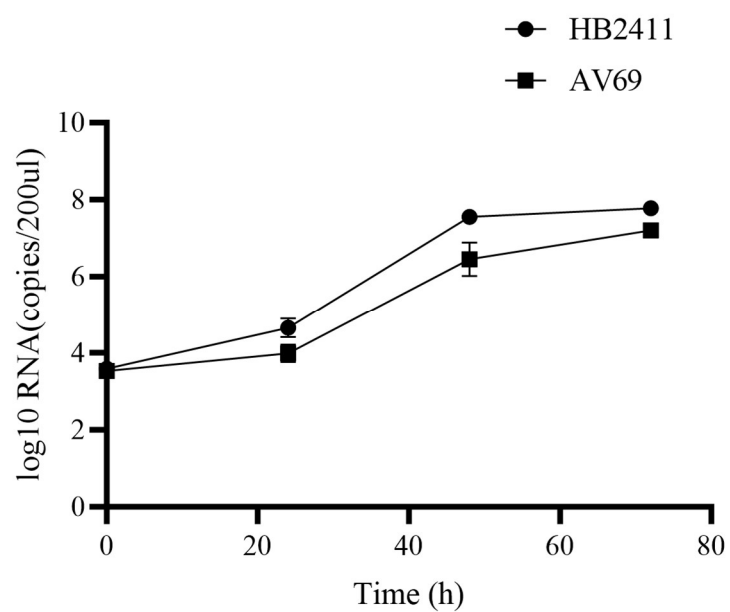

**Figure S1.** Copy numbers of strains HB2411 and AV69 in infected primary liver cells.

Supplement: Supplementary file 1 [file animals-16-00153-s001.zip › animals-3986921-supplementary.pdf]
